# Personal

**Published:** 1899-11

**Authors:** 


### PERSONAL.

DR. JAMES WRIGHT PUTNAM, of Buffalo, was lately made consulting neurologist to the Craig Colony for epileptics, recently established by the State of New York, at Sonyea. The board of managers have exhibited a progressive spirit in creating a consulting staff, and we congratulate them on this new appointment.

---

DR. WILLIAM HOUSE, of Buffalo, is in the west, where he expects to spend a year in travel, living an out-of-door life, principally on the Pacific slope. He expects to return to Buffalo and resume the practice of his profession early next autumn.

---

DR. JOSEPH H. ROBISON, of Bradford, Pa., University of Buffalo, 1895, and Miss Lettie R. Galbraith, of this city, were married at the Church of the Good Shepherd, October 4, 1899. Dr. Robison was formerly one of the internes at the Erie County Hospital. Dr. and Mrs. Robison will reside at Bradford, Pa.

---

DR. ABRAM T. KERR, adjunct professor and demonstrator of anatomy at the University of Buffalo, is temporarily residing in Baltimore. He will be engaged for some time in special anatomical work at Johns Hopkins University.

